# Biophysical Characterization of Interactions Between Human Salivary Proteins and Quinine

**DOI:** 10.1007/s11483-026-10236-3

**Published:** 2026-10-26

**Authors:** Yashmita Grover, Neela H. Yennawar, John E. Hayes, John N. Coupland

**Affiliations:** 1 Department of Food Science, College of Agricultural Sciences, The Pennsylvania State University, University Park, Pennsylvania USA; 2 Biomolecular Interactions Core Facility, The Huck Institutes of the Life Sciences, The Pennsylvania State University, University Park, Pennsylvania USA; 3 Sensory Evaluation Center, College of Agricultural Sciences, The Pennsylvania State University, University Park, Pennsylvania USA

**Keywords:** Salivary proteins, Quinine, Protein-bitterant binding, Bitter taste, Sedimentation velocity analytical ultracentrifugation, Isothermal titration calorimetry

## Abstract

Saliva is the first biological medium encountered by bitter compounds in the oral cavity, and salivary proteins have been indirectly implicated in bitterness perception. However, direct biophysical characterization of salivary protein-bitterant interactions has not been reported. This study investigated interactions between salivary proteins and the model bitterant quinine using complementary biophysical techniques. Pooled whole saliva (WS) from 44 healthy volunteers was separated into high- (HMW) and low-molecular-weight (LMW) fractions using a 3 kDa filter. Protein-quinine interactions were characterized by fluorescence spectroscopy, sedimentation velocity analytical ultracentrifugation (SV-AUC), and isothermal titration calorimetry (ITC). Fluorescence spectroscopy showed a concentration-dependent reduction in the apparent unbound quinine, reaching 59.28 ± 0.89% at 0.6 mg/mL protein in WS, with greater apparent binding in the LMW fraction than the HMW fraction. SV-AUC provided evidence of protein-quinine complex formation, with the LMW fraction exhibiting the clearest changes through the appearance of new, higher sedimentation coefficient species, suggesting quinine-induced multimerization. ITC revealed endothermic binding across all three saliva systems, with the LMW fraction exhibiting the greatest change in apparent binding enthalpy (∆H = 1.053 ± 0.009 kcal/ mol) compared with WS (∆H = 0.321 ± 0.548 kcal/ mol) and HMW fraction (∆H = 0.081 ± 0.158 kcal/ mol). These findings converge to demonstrate that human salivary proteins interact with quinine, with the LMW species (< 3 kDa) exhibiting the strongest apparent binding (17.53 ± 1.58% reduction in unbound quinine at 0.2 mg/mL protein from the fluorescence measurements). This work provides the first biophysical characterization of salivary protein-bitterant interactions and establishes a foundation for future investigations into the role of salivary proteins in bitterness perception.

**Graphical Abstract:**

**Figure:**
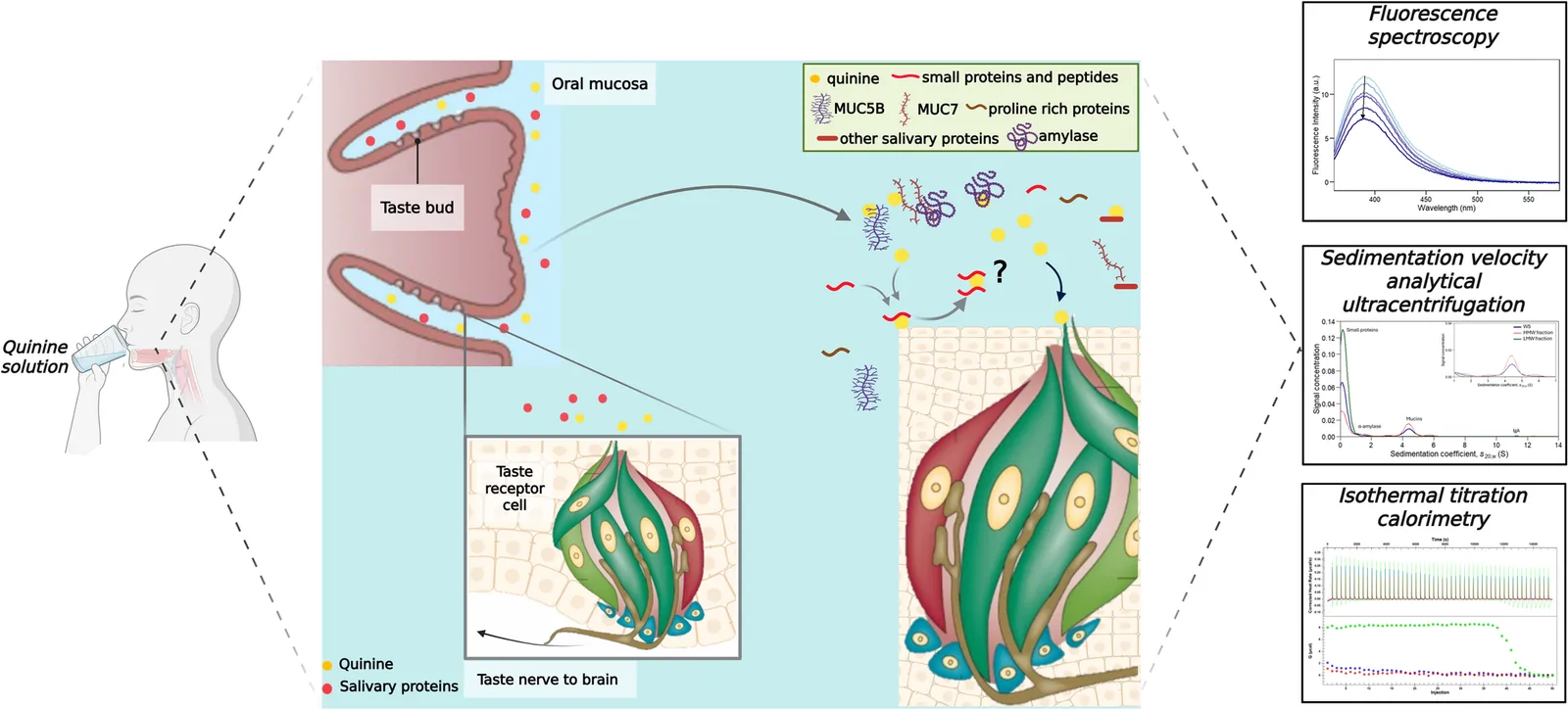

## Introduction

The perception of taste requires the release of taste-active compounds from food and their interaction with cognate receptor cells. However, before the tastant can interact with its receptor, it first encounters saliva.

Water constitutes approximately 99% of saliva, while the remaining fraction is comprised of a complex mixture of fatty acids, electrolytes, and a diverse array of proteins and peptides [1]. The proteinaceous component alone encompasses more than 5,500 different species, including mucins, α-amylase, cystatins, proline-rich proteins (PRPs), among many others [2]. This protein repertoire is predominantly synthesized and secreted by the three major pairs of salivary glands (submandibular, sublingual, and parotid), with additional contributions from other systems and organs including oral epithelial cells, the bloodstream, and the oral microbiome [3]. The compositional complexity of the salivary proteome is further amplified within the oral cavity, where proteins undergo mixing, post-translational modification, and proteolytic cleavage by endogenous and microbial enzymes, generating a heterogeneous molecular environment.

Various studies have implicated salivary proteins in the perception and modulation of oral sensations. Their involvement in astringency perception has been particularly well documented, with PRPs [4] and mucins [5] being the most frequently implicated species. The interaction of astringent compounds, such as tannins and other polyphenols, with salivary and oral mucosal proteins, followed by protein precipitation and aggregation, disrupts the salivary pellicle and reduces oral lubrication. The resulting increase in oral friction and mechanical stimulation of chemo- and mechanoreceptors are thought to contribute to the perception of astringency [6].

Beyond astringency, correlational evidence has linked salivary protein composition to taste and flavor perception, with associations reported for mucins [7, 8], proline-rich proteins (PRPs) [9–11], α-amylase [12–15], cystatins [16], statherin [17], prolactin inducible proteins [11, 18], von Ebner’s gland protein [19], and carbonic anhydrase VI [20–22].

Bitter taste is one of the five prototypical taste modalities and serves an important protective function by enabling the detection of potentially harmful compounds, often eliciting aversive reactions. The potential involvement of salivary proteins in bitter taste perception and acceptance has attracted research interest, and several candidate proteins have been identified through correlational studies. Higher salivary levels of the basic PRP peptides Ps-1 and II-2 have been reported in individuals more sensitive to the bitter taste of 6-n-propylthiouracil (PROP) compared to non-tasters [9, 10]. Conversely, elevated levels of cystatins have been associated with hyposensitivity to caffeine bitterness in adults [12] and with greater acceptance to bitter urea solutions in infants [18]. More recently, salivary glutathione transferases have been proposed to modulate bitter taste through direct interactions with bitterants in vitro [23]. Evidence from rodent models further supports a role for salivary proteins in bitter taste adaptation, as changes in salivary protein profiles have been correlated with increased acceptance of novel bitter-containing diets following 14 days of repeated dietary exposure [24].

Despite this growing body of evidence, the mechanisms by which salivary proteins influence bitterness perception remain speculative, as most studies have relied on correlation rather than direct mechanistic investigation. One proposed mechanism involves direct interactions between salivary proteins and bitter stimuli, leading to the formation of protein-bitterant complexes that may modulate bitterness either by sequestering the stimulus and reducing its availability for receptor binding [25], or by facilitating the interaction of bitterants with bitter taste receptors [26, 27]. In contrast to the extensive literature characterizing salivary protein-polyphenol interactions in astringency perception using biophysical techniques, the direct characterization of interactions between salivary proteins and pure bitter compounds has, to our knowledge, not been reported previously. Bridging this gap requires the application of complementary biophysical techniques capable of detecting and characterizing protein-ligand interactions under physiologically relevant conditions.

Quinine, an alkaloid derived from the bark of *Cinchona* tree, is widely used in psychophysical research as a model compound because of its characteristic bitter taste, absence of other side tastes, and widespread use in food and beverage systems. It has also been extensively employed in studies investigating bitter taste perception [28, 29]. In the present study, quinine was used as a model stimulant to investigate salivary protein-bitterant binding interactions. Specifically, this study aimed to: (1) evaluate quinine binding to salivary proteins and quantify the concentration of unbound quinine using fluorescence spectroscopy; (2) characterize the salivary protein populations involved in binding interactions with quinine by evaluating changes in sedimentation behavior using sedimentation velocity analytical ultracentrifugation (SV-AUC); and (3) determine the thermodynamic characteristics of salivary protein-quinine interactions using isothermal titration calorimetry (ITC). The present study focused on the physicochemical interactions between salivary proteins and quinine as a foundational step for subsequent work that may include psychophysical testing to evaluate their effect on bitterness perception, receptor activation, or on the effective concentration of quinine available for receptor binding.

Drawing on the reported interactions of salivary proteins with sensory stimuli such as polyphenols, as well as the associations between salivary protein composition and sensitivity to bitter taste, this study tests the hypothesis that human salivary proteins interact with quinine and that the magnitude and physicochemical characteristics of these interactions differ among salivary protein populations.

By providing the first biophysical characterization of interactions between human salivary proteins and quinine, this work provides a mechanistic insight into salivary protein-bitterant interactions and seeks to establish a strong foundation for future investigations into the role of salivary proteins in modulating taste perception in humans.

## Materials and Methods

### Ethics Statement

Panelist recruitment, screening, and all study procedures were approved by the Pennsylvania State University’s Institutional Review Board (protocol number: STUDY00027802). In compliance with the Declaration of Helsinki, informed consent was obtained from all participants prior to their involvement in the study procedures, and they were informed of their right to withdraw from the study at any point in time without consequence.

### Participant Recruitment and Consent

Participants (*n* = 44) for the study were recruited from The Pennsylvania State University campus and the surrounding region (State College, PA, USA). Participant screening, recruitment, and saliva collection were conducted as a part of a concurrent study being carried out in the Sensory Evaluation Center in the Erickson Food Science Building, on chewing efficiency. The eligibility criteria required that potential participants: be between 22 and 60 years of age; be fluent in English; not wear full or partial dentures routinely during the day; have no prior diagnosis of an eating disorder; not be pregnant or breastfeeding at the time of the study; not have taken any medications known to alter appetite, smell, or taste in the last three months; not be taking prescription pain medications; not be users of nicotine-containing products; have no history of choking or difficulty swallowing; have no tongue, lip or cheek piercings; have no issues with taste or smell; have no history of pain in the mouth, or face, or discomfort during jaw movements; not have undergone dental work within the past 30 days; have no chewing disabilities; and possess a full set of natural teeth. No bitterness intensity ratings or other psychophysical measures related to bitter taste perception were collected as part of the present study. Upon completion of their study session, participants were compensated for their time with a cash incentive.

### Saliva Collection and Processing

Prior to saliva collection, all participants were made to consume 4 ounces of water to help standardize hydration levels across individuals. Saliva was then collected following a procedure adapted from Ma et al. [30]. Briefly, participants were asked to provide a parafilm-stimulated saliva sample by chewing on a 5 × 5 cm piece of Parafilm (Parafilm “M”, American National CAL, Chicago, IL, USA) for 5 min, spitting intermittently into a 50 mL sterile conical centrifuge tube every 30 s; tubes were maintained on ice to ensure temperature control. Immediately following collection, a protease inhibitor cocktail (Halt™ Protease Inhibitor Cocktail,100X, EDTA-free, Thermo Scientific, Waltham, MA, USA; containing inhibitors at concentrations: AEBSF, 1 mM; Aprotinin, 800 nM; Bestatin, 50 µM; E64, 15 µM; Leupeptin, 20 µM; and Pepstatin A, 10 µM) was added at 10 µL/mL as per manufacturer specifications, to inhibit protein degradation by endogenous salivary proteases [31]. Saliva samples were maintained on ice and processed within 3 h of collection. Processing involved centrifugation at 2,000 X *g* for 6 min to pellet cellular debris and other insoluble material [32]; the pellet was discarded and the clarified supernatants from individual samples were pooled in a glass jar and stored at 4 °C until saliva samples had been collected from all the recruited participants across the 3-day collection period. Short term storage at 4 °C for up to 2 days was not expected to compromise protein integrity, given the addition of protease inhibitors; Chevalier et al. [31] reported no detectable protein degradation in one-dimensional profiles of human saliva samples (with no added protease inhibitors) stored at 4 °C for up to 30 days. Following completion of saliva collection and centrifugal clarification, all samples were pooled together, and the representative human saliva sample thus obtained was designated “whole saliva” (WS) for the remainder of this study. The pH of the pooled WS was measured, after which it was aliquoted into 1 mL and 10 mL volumes and stored at -80 °C. For analysis, samples were thawed in the refrigerator at 4 °C.

### Saliva Fractionation

Considering the compositional complexity and heterogeneity of salivary proteins and peptides, WS was fractionated into high- and low-molecular-weight salivary protein populations to better evaluate their respective contributions to quinine binding. A 3 kDa molecular weight cutoff filter was selected as a size-based approach to separate the majority of higher-molecular-weight salivary proteins from lower-molecular-weight proteins and peptides, following a fractionation approach used by Martin et al. [33] to investigate the role of salivary proteins in taste nerve signaling in rats. Fractionation was performed using Amicon^®^ Ultra 15 mL Centrifugal Filter Units (3 kDa MWCO, UFC9003, MilliporeSigma, Burlington, MA, USA). Filter preparation and protein fractionation were carried out by centrifugation according to manufacturer-specified conditions. Briefly, the filter was first conditioned by centrifugation at 4,000 X *g* for 10 min at 4 °C using 12 mL of ultrapure water (generated by Milli-Q Plus system, Millipore Corp., Bedford, MA, USA). Following conditioning, the water was discarded and 12 mL of thawed WS was loaded into the filter and centrifuged at 4000 X *g* for 40 min at 4 °C, reducing the sample volume to less than 2 mL. The filtrate, containing proteins and peptides < 3 kDa, is hereafter referred to as the “low-molecular-weight fraction” (LMW fraction), and the retentate, containing proteins > 3 kDa, is hereafter referred to as the “high-molecular-weight fraction” (HMW fraction).

It should be acknowledged here that the 3 kDa cutoff represents an operational rather than an absolute separation boundary. As noted by Singh [33], centrifugal membrane filters if this type are subject to inherent limitations, including non-uniform pore size distributions across the membrane, formation of protein films at the membrane surface, and influence of protein shape and conformation on retention behavior. These factors can result in some overlap in the molecular-weight distribution of proteins in the retentate and filtrate fractions. Accordingly, the HMW and LMW fractions obtained in study should be considered as fractions enriched in higher- and lower-molecular-weight salivary protein constituents, respectively, rather than as strictly separated populations defined by a discrete 3 kDa boundary. The pH and total protein concentration of both fractions were determined on the day of processing; remaining material was aliquoted and stored at -80 °C until further analysis.

### Salivary Protein Quantification and Gel Electrophoresis

Total protein concentration in WS and its fractions was determined by bicinchoninic acid (BCA) assay (Pierce™ BCA Protein Assay Kit, Thermo Scientific, Waltham, MA, USA) following the manufacturer’s instructions. Reagents A and B were freshly combined in a 50:1 ratio (v/v) to prepare the working reagent. In a 96-well plate, 25 µL of each saliva sample was combined with 200 µL of the working reagent, briefly agitated on an orbital shaker at 600 rpm for 30 s, and then incubated at 37 °C inside the microplate reader. Absorbance was subsequently measured at 562 nm using a Thermo Scientific Multiskan Go microplate spectrophotometer (Thermo Fisher Scientific, Vantaa, Finland). Protein concentrations were determined in triplicate against a bovine serum albumin (BSA) standard curve (0–2000 µg/mL; 9 working concentrations; BSA stock concentration: 2 mg/mL). Given the heterogeneous protein composition of saliva and its fractions and the use of BSA as the calibration standard, the reported protein concentrations were considered apparent estimates rather than absolute concentrations of the proteins and peptides present in the samples.

Sodium dodecyl sulfate-polyacrylamide gel electrophoresis (SDS-PAGE) was performed according to the protocol previously described by Martin et al. [34]. Briefly, equal volumes of saliva samples were mixed with one-third volume of 4X NuPAGE™ LDS sample buffer (Life Technologies, Carlsbad, CA) with a reducing agent and heated at 85 °C for 10 min. Sample volumes containing equivalent protein concentrations were resolved on a precast 12% Criterion™ XT Bis-Tris gel (Bio-Rad Laboratories, Hercules, CA) using 3-(N-morpholino)propanesulfonic acid (MOPS) running buffer. A molecular weight marker spanning 10–250 kDa (Precision Plus Protein™ Dual Color Standards, Bio-Rad Laboratories, Hercules, CA) was run alongside the samples to provide molecular weight references for the observed protein bands. Following electrophoresis at a constant voltage of 200 V for 1 h, the gels were fixed for 30 min using gel-fixing fixing solution containing methanol, acetic acid, and ultrapure water (50:10:40 v/v/v), followed by staining with Coomassie Brilliant Blue (CBB) R-250 (0.1% CBB R-250 in 30% methanol, 5% acetic acid, and 65% ultrapure water, v/v/v) for 1.5 h. The gels were destained overnight by incubation in 10% (v/v) acetic acid solution. Protein bands on the gel were visualized using GelDoc Go Gel Imaging System (Bio-Rad Laboratories, Hercules, CA).

### Preparation of Saliva-quinine Solutions

Quinine monohydrochloride dihydrate was purchased from Sigma-Aldrich (St. Louis, MO, USA). Ultrapure water was used throughout all experiments. Saliva-quinine solutions were prepared following the protocol described in an earlier paper from our laboratory for the preparation of protein-quinine solutions [35]. A series of saliva dilutions were prepared from each saliva sample (WS, HMW fraction, and LMW fraction) using ultrapure water to obtain reaction mixtures spanning a range of salivary protein concentrations. An aqueous quinine stock solution (5000 ppm) was then added to each mixture to achieve a final quinine concentration of 17 ppm (0.042 mM). Phosphate buffer (0.05 M), prepared at a pH corresponding to that of the respective saliva sample (WS, HMW fraction, or LMW fraction), was subsequently added in equal mass across all samples to yield a final buffer concentration of 0.01 M in the mixture. The maximum salivary protein concentration for each series of reaction mixtures was determined by the measured total protein concentration of the respective saliva sample while maintaining a constant total reaction mixture mass and a fixed quinine concentration of 17 ppm. Accordingly, WS mixtures contained salivary protein concentrations ranging from 0 to 0.6 mg/mL, whereas reaction mixtures containing the LMW fraction ranged from 0 to 0.2 mg/mL, based on the maximum protein concentration attainable in the LMW fraction. Consequently, to facilitate direct comparison in quinine-binding ability between the fractions, the HMW fraction was also evaluated over the same protein concentration range. The mixtures were prepared in opaque brown glass containers, and immediately following preparation, stirred at 500 rpm for 20 min at room temperature to facilitate salivary protein-quinine interactions and complex formation. All subsequent analyses were conducted immediately after the incubation period. The quinine concentration of 17 ppm was selected because it corresponds to a bitterness intensity between weak (6) and intermediate (17) on a general Labeled Magnitude Scale (gLMS) [36], based on previously reported dose-response functions [37]. This concentration was therefore considered representative of a practically relevant bitterness intensity for future studies involving physicochemical binding analyses along with sensory evaluation.

### In Vitro Analysis of Salivary Protein-quinine Interactions

#### Fluorescence Quenching

Interactions between salivary proteins and quinine were investigated using fluorescence spectroscopy, specifically by studying quinine fluorescence quenching in the presence of increasing salivary protein concentrations. Intrinsic fluorescence emission spectra of quinine were recorded at room temperature using an Agilent Cary Eclipse Fluorescence Spectrophotometer (Agilent Technologies, Inc., Santa Clara, CA, USA). A UV Quartz micro fluorescence cuvette (FireflySci, Inc., Northport, NY, USA) with a sample volume of 0.7 mL and light path of 10 mm was used to record emission spectra from 360 to 580 nm, with an excitation wavelength of 347 nm. Excitation and emission slit widths were set at 2.5 nm, and spectra were acquired at a scan speed of 120 nm/min. For each sample, background signals were subtracted using blanks consisting of buffer alone or buffer containing protein at the corresponding concentration.

Calibration curves for quinine were constructed by measuring its fluorescence intensity across a concentration range of 0–25 ppm in buffered solutions prepared at the pH corresponding to each respective saliva sample. Use of separate buffers was required as quinine fluorescence is pH dependent. The fluorescence emission maxima of quinine were observed at 390 nm at pH 7.98 and 8.33, and at 388.93 nm at pH 8.03, corresponding to the conditions for the WS, HMW fraction, and LMW fraction, respectively. These calibration curves were subsequently used to estimate the concentration of quinine remaining in the salivary protein-quinine mixtures from the measured fluorescence intensity. Spectroscopy-based calibration approaches of this kind have been used in previous studies to estimate unbound ligand concentrations [38–40]. However, it should be acknowledged that this approach relies on the assumption that fluorescence response of quinine in the salivary matrix is equivalent to that in the calibration buffer, and fluorescence intensity changes arise solely from a reduction in the concentration of free quinine rather than from other photophysical phenomena such as interactions with non-protein salivary constituents, or changes in quinine protonation state. Because the fluorescence characteristics of unbound quinine in the salivary protein-quinine mixtures were not independently established, and because the salivary matrix may influence the fluorescence response, the values derived from this approach represent apparent rather than absolute estimates of the unbound quinine concentration. Accordingly, throughout this manuscript, quinine concentrations estimated from fluorescence measurements should be interpreted as fluorescence-estimated apparent unbound quinine concentration. All experiments were performed in triplicate.

#### Sedimentation Velocity Analytical Ultracentrifugation (SV-AUC)

Saliva-quinine mixtures were prepared as described above (0.2 mg/mL protein + 17 ppm quinine in phosphate buffer at the respective saliva sample pH) and loaded into 12 mm titanium centerpieces within AUC cells fitted with sapphire windows. A total protein concentration of 0.2 mg/mL was used for all saliva samples and saliva-quinine mixtures, corresponding to the maximum protein concentration achievable from the LMW fraction. Blanks containing saliva alone (0.2 mg/mL protein) or quinine alone (17 ppm) in a buffered system were run in parallel. Loaded cells were placed into a 4-hole AN-60 titanium rotor, which was then placed inside the chamber of a Beckman Coulter Optima multiwavelength analytical ultracentrifuge (Indianapolis, IN, USA) equipped with multiwavelength absorbance optics. The chamber was evacuated to complete vacuum, and the rotor loaded with samples was allowed to equilibrate at 20 °C for 2 h prior to the start of the run. An experimental method scan was designed in UltraScan III software and transferred to the Beckman Optima instrument; experiment run and data acquisition commenced upon completion of temperature equilibration. The rotor was operated at 60,000 rpm for 23 h under full vacuum at 20 °C, with radial absorbance scans acquired every 2 min 50 s at 280 nm. The extended run time was used to allow sufficient migration of slowly sedimenting, LMW salivary constituents.

Upon completion of the AUC run, raw radial intensity data were imported into UltraScan III [41] and converted to pseudo-absorbance by manual selection of reference scans. The air-liquid meniscus was identified manually for each sector, and the data range was set from ~ 6.1 to 7.1 cm. Initial (5–10) and terminal (5–10) scans following complete sedimentation, which lacked a meaningful change in signal concentration, were excluded from the data. The cleaned dataset was subsequently processed on the LIMS supercomputer at Penn State using an S-Value range of 0–14 with a resolution of 100, and a frictional ratio range of 1–4 with a resolution of 64. Time-invariant noise was accounted for during the initial 2D spectral analysis. Once residuals fell below 0.003, the data were refitted for both time- and radially invariant noise, with simultaneous meniscus fitting to ensure precise determination of the meniscus position. Following identification of the correct meniscus, a final fit for time and radially invariant noise was performed using an iterative approach with a maximum of 10 iterations [42]. Sedimentation velocity data were ultimately analyzed using continuous c(s) processing, which generates sedimentation coefficient distributions, s_20,w_ (expressed in Svedberg units; S = 10^− 13^ s) by fitting experimental boundary profiles to numerical solutions of the Lamm equation [43].

#### Isothermal Titration Calorimetry (ITC)

The binding enthalpy associated with the interaction of quinine and salivary proteins was determined using an Affinity ITC calorimeter (TA Instruments, New Castle, DE, USA) equipped with an 185 uL active volume cylindrical gold reaction cell. The quinine concentration and ITC operating parameters were selected based on prior experiments characterizing protein-quinine interactions done by our laboratory which demonstrated sufficient heat signal and facilitated direct comparison across experiments. All solutions were prepared in ultrapure water. ITC experiments were not conducted in buffered solutions because quinine, at the experimental concentration and temperature conditions involved in the study, was insoluble in the buffer. For each measurement, 185 µL of saliva sample at 0.2 mg/mL protein was loaded into the calorimetric cell thermostatted to 25 °C and titrated with 8 mM quinine via successive 1 µL injections with continuous stirring at 200 rpm. The interval between injections was 300 s, with each injection lasting 20 s. A blank titration of quinine into ultrapure water was performed to determine the heat of dilution, which was subsequently subtracted from the raw data. Since this correction accounts only for the heat of dilution of quinine and does not correct for potential contributions from quinine protonation, differences in ionic environment between the syringe and cell constituents, or other non-proteinaceous components of the salivary matrix, the measured enthalpies are therefore to be interpreted as apparent values reflecting the net thermodynamic response from the system and not as exclusive contributions from salivary protein-quinine interactions. Human saliva is a heterogeneous biological fluid containing thousands of proteins and peptides that vary substantially in concentration and molecular weight [44]. Consequently, saliva does not possess a single, well-defined molecular weight that can be used to calculate molar concentration. Because binding models in NanoAnalyze software (TA Instruments, New Castle, USA) require the molar concentrations of the interacting protein components as inputs, no predefined binding model could be applied during data analysis. 

### Data Analysis

Data analyses were performed in RStudio (Version 2026.5.1.225; Posit Software, PBC, Boston, MA, USA) using R (Version 4.4.1). Salivary protein-quinine binding data were analyzed using linear regression models. Figures were made in RStudio.

## Results and Discussion

### Salivary Protein Concentration, pH, and Electrophoretic Profile

The measured total protein concentrations and pH values of the saliva samples are presented in Table 1. The total protein concentration of WS was within the range previously reported for healthy individuals (0.5-2.0 mg/mL) [45]. Following fractionation using the 3 kDa MWCO filter, the HMW and LMW fractions had higher and lower total protein concentrations than WS, respectively. This distribution is consistent with retention of the majority of salivary proteins in the HMW fraction during filtration.

The pH of the WS was 7.98. Resting human saliva typically has a pH between 6.2 and 7.6, although higher values, up to approximately 8.1 units, have been reported under stimulated conditions due to increased salivary flow rate [46–47]. The pH observed for WS in the present study was therefore slightly higher than the typical resting range but falls within the reported range for stimulated saliva. The differences in pH among WS and its HMW and LMW fractions may reflect differences in their protein and ionic composition following fractionation.

**Table 1:** Total protein concentration and pH of the saliva samples

Saliva sample	Total protein concentration (mg/mL)	pH
Whole saliva	0.99 ± 0.03	7.98
HMW fraction	2.07 ± 0.06	8.33
LMW fraction	0.26 ± 0.02	8.03

**Fig. 1:**
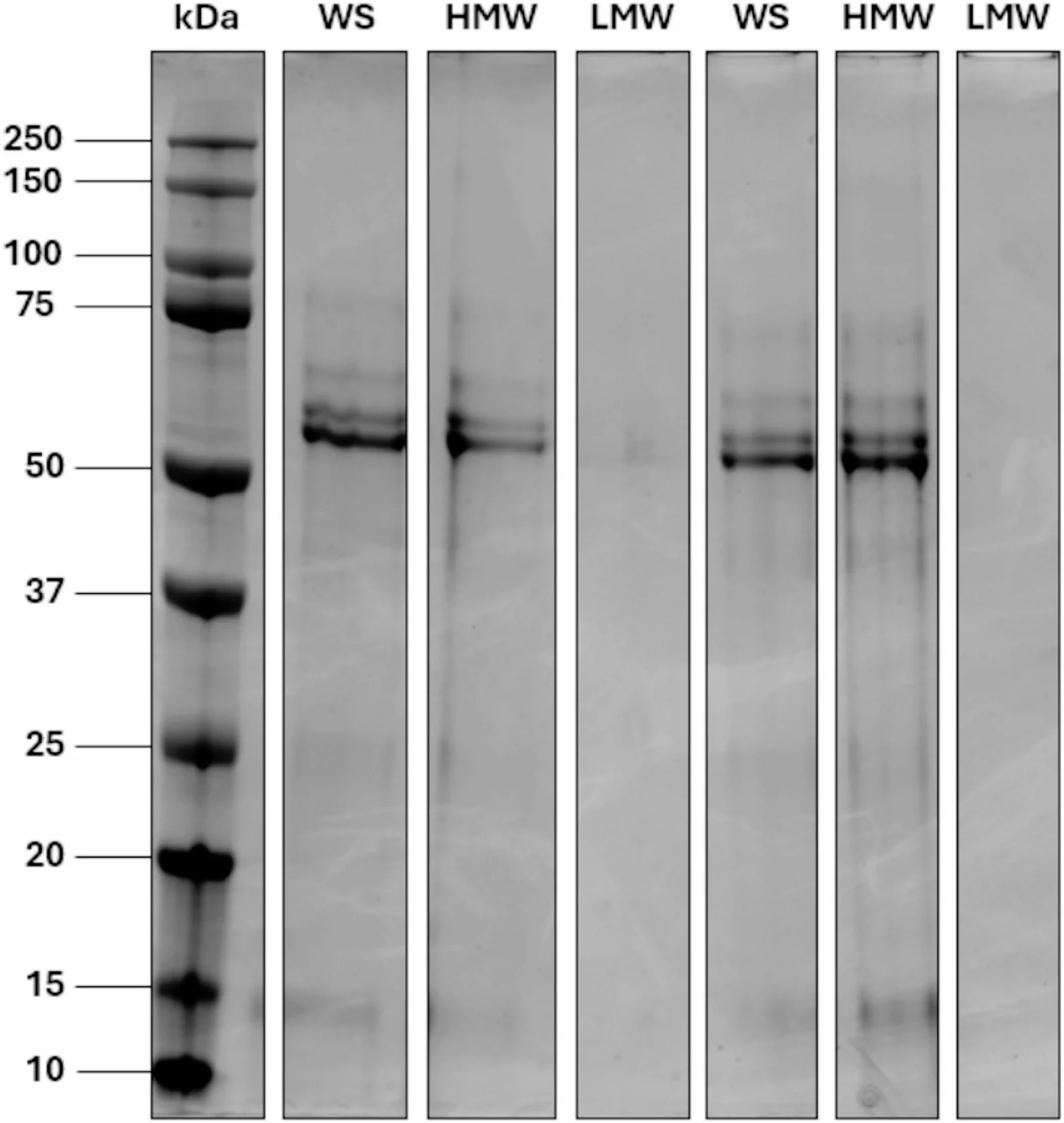
One-dimensional SDS-PAGE separation of salivary proteins using equal protein loading (1.85 µg per lane). Lanes represent the molecular weight marker (kDa), whole saliva (WS), high-molecular-weight (HMW) fraction, and low-molecular-weight (LMW) fraction, with each saliva sample run in duplicate

To qualitatively compare the protein composition of WS and its fractions, SDS-PAGE analysis was performed. Under the electrophoretic conditions employed, WS and the HMW fraction exhibited similar protein banding patterns (Fig. 1), with multiple bands spanning a broad molecular weight range and prominent bands between 50 and 75 kDa, consistent with those previously reported for human saliva [31, 48]. No discrete bands were observed for the LMW fraction. This observation is consistent with the predominance of proteins and peptides smaller than 3 kDa in the LMW fraction, as well as with the limited sensitivity of conventional SDS-PAGE for visualization of proteins smaller than ~ 10 kDa [49, 50].

### Fluorescence Quenching Analysis

The apparent binding interactions between salivary proteins and quinine, were assessed by quantifying changes in quinine fluorescence intensity following the addition of salivary protein solutions.

**Fig. 2:**
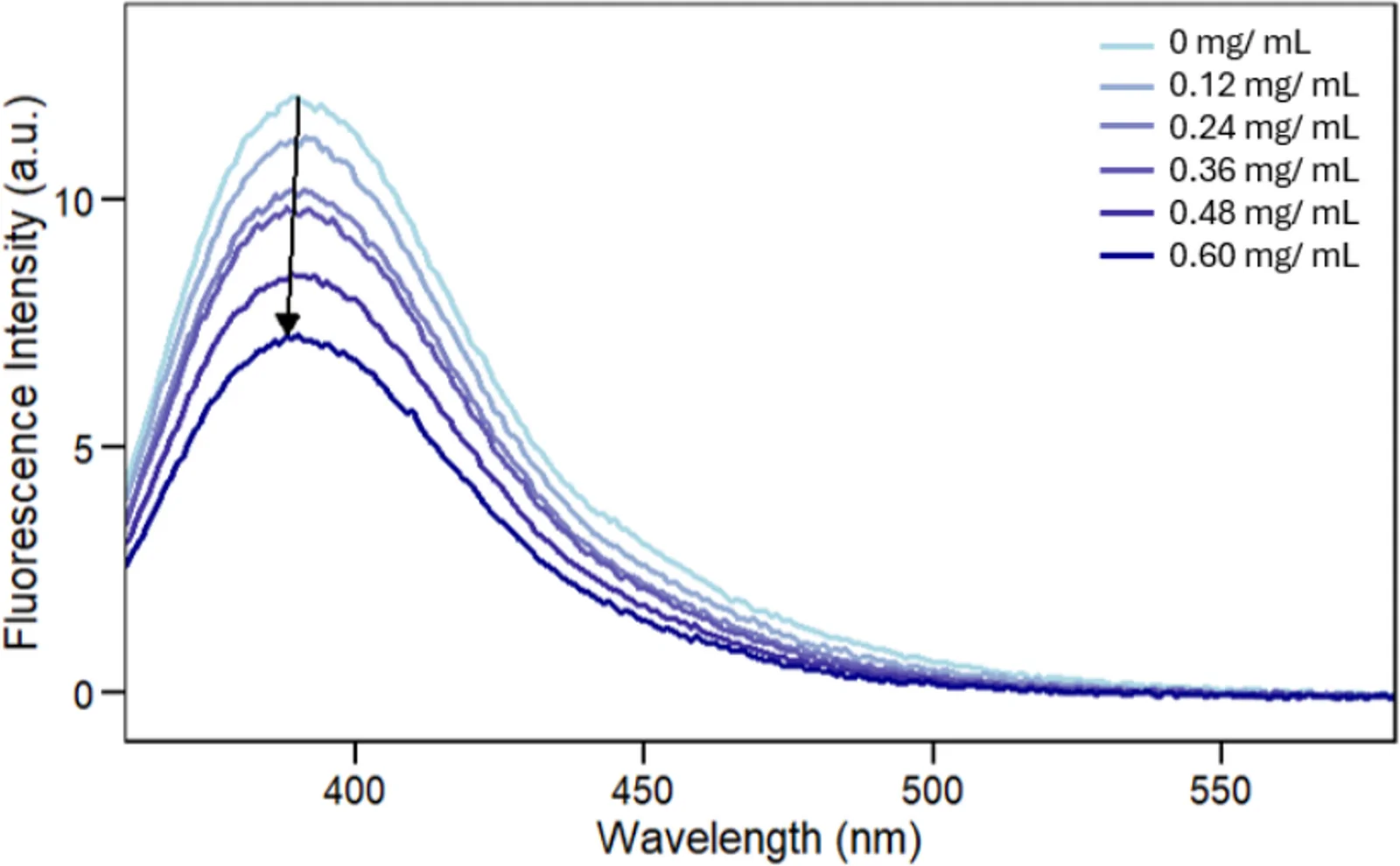
Fluorescence emission spectra (at λ_ex_ = 347 nm) of quinine (17 ppm) in the presence of increasing concentrations of whole saliva proteins (0-0.6 mg/mL). Each curve represents the mean values of a triplicate assay after correction for buffer and saliva fluorescence. The arrow indicates the overall direction of the shift in the emission maxima of quinine between 0 mg/mL and 0.6 mg/mL whole saliva protein concentrations

Figure 2 shows the fluorescence emission spectra of quinine (λ_em_ = 360–580 nm, λ_ex_ = 347 nm) as a function of WS concentrations (0-0.6 mg/mL protein; pH = 7.98). Increasing WS concentration resulted in a decrease in quinine fluorescence intensity, suggesting binding interactions between salivary proteins and quinine. Concomitantly, a small blue shift in the emission maximum of quinine (from 390 nm to 388 nm) was observed at protein concentrations ≥ 0.36 mg/mL, indicating a change in the immediate microenvironment surrounding quinine upon interaction with salivary proteins. A blue shift in fluorescence emission is generally attributed to a reduction in the polarity of the fluorophore’s surrounding environment [51]. In the present study, this observation may indicate that quinine transitions into a relatively less polar, more hydrophobic microenvironment upon interaction with salivary proteins, consistent with its hydrophobic nature and the potential involvement of hydrophobic interactions in the binding process. A similar blue-shift in quinine emission fluorescence spectrum accompanied by a reduction in fluorescence intensity was reported by Datta et al. [52] following titration of Q1 aptamer (1µM) into quinine (50 nM), which the authors attributed to direct interactions between the two molecules.

**Fig. 3:**
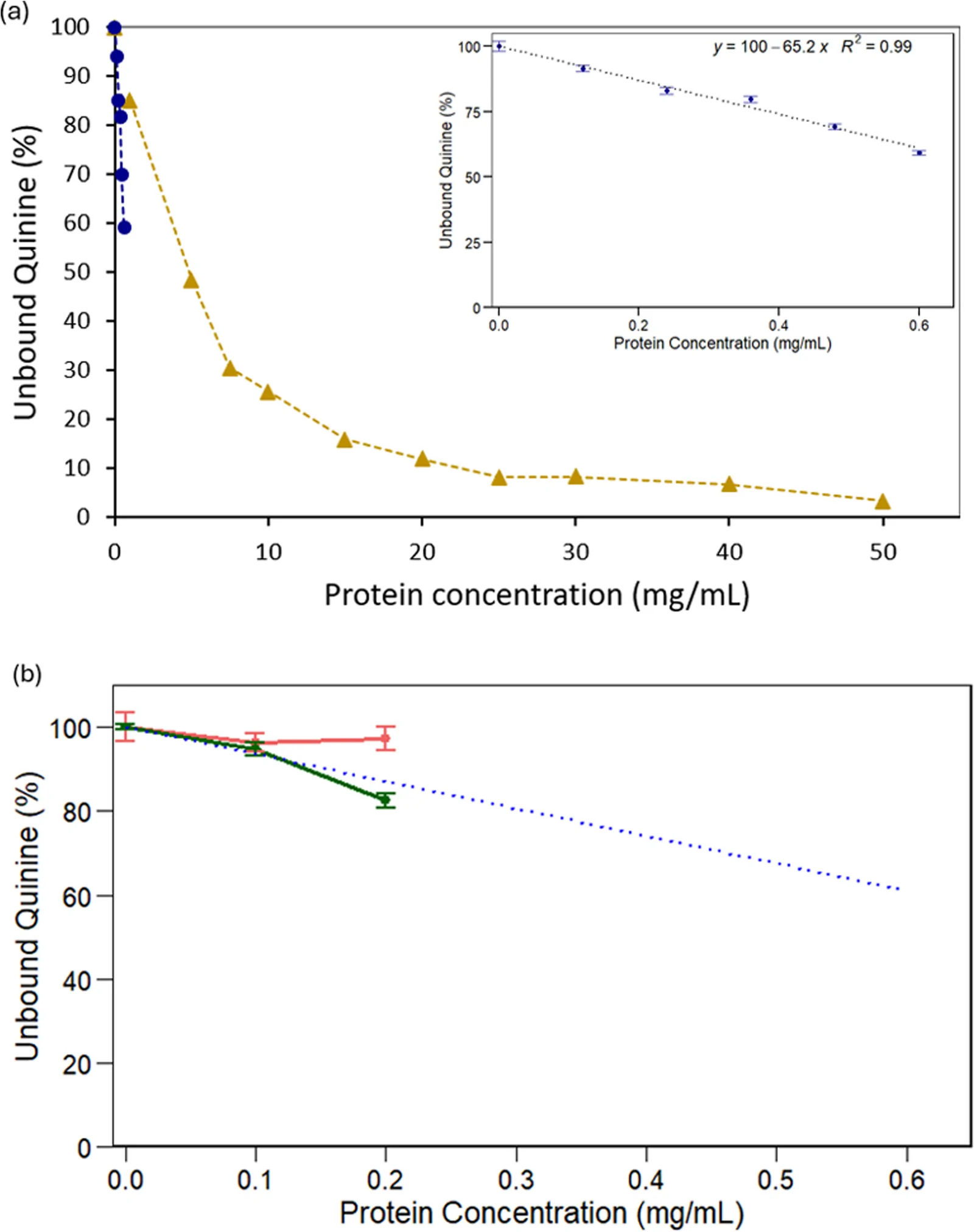
Protein-quinine binding. (**a**) Proportion of unbound quinine (total concentration 17 ppm) as a function of increasing protein concentration in whole saliva (WS; blue circles, 0-0.6 mg/mL) and sodium caseinate (NaCas; yellow triangles, 0–50 mg/mL). NaCas data were adapted from Grover et al. [35]. The insert shows the WS binding data with the fitted linear regression binding model (dotted line) and the corresponding regression equation and R^2^ value. (**b**) Proportion of unbound quinine (total concentration 17 ppm) as a function of increasing protein concentration in the HMW (red circles, 0-0.2 mg/mL) and LMW (green circles, 0-0.2 mg/mL) fractions. The blue dotted line represents the fitted linear regression model for WS shown in (**a**). Error bars indicate standard deviation

The percentage of unbound quinine, calculated from the proportional reduction in fluorescence intensity, is shown as a function of protein concentration in Fig. 3a (blue curve). At the maximum protein concentration evaluated (0.6 mg/mL), the measured quinine fluorescence intensity decreased from 12.11 ± 0.155 a.u. in the absence of salivary proteins to 7.276 ± 0.052 a.u. (39.92 ± 0.88% reduction). Using the quinine calibration curve for estimating the concentration of quinine in the unbound form, these fluorescence intensity values corresponded to a 40.72 ± 0.89% reduction in the apparent unbound quinine, with 59.28 ± 0.89% of the quinine remaining unbound, and thus indicating substantial binding of quinine by salivary proteins. A linear regression model provided an excellent fit to the WS binding data (R^2^ = 0.99; Fig. 3a insert), yielding an empirical binding coefficient of 65.2% reduction in the unbound quinine per mg/mL protein. A previous study from our laboratory investigating the binding interactions between quinine (17 ppm) and the dietary protein sodium caseinate (NaCas; 0–50 mg/mL) also observed a reduction in unbound quinine concentration with increasing protein concentration. In that study, an empirical logarithmic model provided a good fit to the NaCas-quinine binding data ($\:y=\:-21.3\mathrm{ln}\left(x\right)+30.49$; R^2^ = 0.96). In the present study, a linear model was used for the WS data because of the narrower protein concentration range examined and the excellent model fit obtained. When the quinine-binding profiles of NaCas and WS were plotted together (Fig. 3a), the proteinaceous components of WS achieved the same reduction in unbound quinine concentration at approximately 4.3-fold lower protein concentration than NaCas. While differences in experimental conditions, including pH and mixture preparation, must be acknowledged, these observations do suggest a substantially greater quinine-binding ability of salivary proteins under the studied conditions.

Binding was also assessed using the HMW and LMW fractions over a protein concentration range of 0-0.2 mg/mL (Fig. 3b). Because only two non-zero protein concentrations were examined, it was not considered appropriate to fit a model to the data. Relative to quinine alone under the respective experimental conditions, the addition of both saliva fractions resulted in a reduction in the proportion of unbound quinine. At a protein concentration of 0.2 mg/mL, 97.23 ± 2.78% and 82.47 ± 1.58% of the quinine remained unbound in the HMW and LMW fractions, respectively (while the corresponding value for WS was 86.96%). This reduction in the proportion of unbound quinine was small for the HMW fraction compared with the LMW fraction at the same protein concentration, indicating greater quinine binding in the LMW fraction. Overall, these findings indicate both saliva fractions interacted with quinine, with greater apparent binding observed for the LMW fraction than the HMW fraction, while WS showed an intermediate response. Several factors may contribute to the greater apparent quinine binding observed for the LMW fraction, including differences in protein composition, molecular weight, structural accessibility of binding regions, and the non-protein composition of the fractions. Differences in solvent conditions, including pH and ionic strength, may also contribute to the observed differences in binding between the fractions. First, the LMW fraction contains a heterogeneous mixture of proteins and peptides smaller than 3 kDa in size, many of which are generated through the proteolytic processing of salivary proteins, including statherins, proline-rich proteins, and histatins, by salivary and microbial proteases [53]. These small proteolytic products would be excluded from the HMW fraction during fractionation. Furthermore, Perinpanayagam et al. [53] identified small histatin-derived peptides (< 3 kDa) in human saliva that retained functional amino acid sequences from the parent proteins involved in hydroxyapatite binding at the tooth surface. They proposed that these fragments may preserve their hydroxyapatite-binding function despite having undergone proteolysis. Although quinine differs considerably from hydroxyapatite in its structural and chemical properties, their findings suggest that proteolytic processing of salivary proteins does not necessarily eliminate the ligand binding ability of the resulting peptides.

Second, structural differences between the proteins present in the two fractions may also contribute to the observed differences in binding. Small proteins and peptides often exhibit greater conformational flexibility than larger proteins, and many peptides below 3 kDa are partly or predominantly unstructured in solution. This may increase the accessibility of amino acid residues capable of participating in binding interactions with quinine, whereas in larger proteins these regions may be more buried or sterically occluded. In addition, several of the major proteins enriched in the HMW fraction exist in extensively glycosylated forms in saliva. The carbohydrate chains associated with these proteins may further reduce the accessibility of potential binding regions. Consistent with this possibility, Lu and Bennick [54] reported that glycosylated PRPs (10 µg) exhibited negligible to no binding to condensed tannins or tannic acid over most of the concentration range examined (0–3 µg/µL), whereas enzymatic deglycosylation using peptide-N-glycosidase resulted in a dramatic increase in binding to both tannin compounds. These findings suggest that protein structure and glycosylation can substantially influence accessibility of some binding regions and may therefore contribute to the comparatively lower quinine binding observed in the HMW fraction as compared to the LMW fraction.

Third, at an equivalent protein concentration on a mass basis, the LMW fraction would contain a greater number of individual protein and peptide molecules than the HMW fraction because of their lower molecular weights. Thus, on a per unit protein mass basis, the LMW fraction may provide a greater number of molecular species available to interact with quinine.

Finally, it should be noted that the HMW and LMW fractions may not have been compositionally equivalent beyond their protein content. In addition to separating the protein constituents based on molecular weight, the 3 kDa filtration step may also have separated other LMW non-protein constituents, including salts, metabolites, and other small molecules. Hence, the saliva solutions prepared from the two fractions may have differed in the concentration of non-protein constituents and their ionic environment.

The present study did not identify the specific proteins and peptides responsible for the observed binding interactions with quinine. Therefore, although the factors discussed above provide plausible explanations for the greater quinine binding observed in the LMW fraction under the studied experimental conditions, definitive identification of the species involved will require complementary protein identification approaches in future studies.

Fluorescence quenching measurements provide evidence of interactions between salivary proteins and quinine through measuring the reduction in quinine fluorescence intensity resulting from changes in its microenvironment upon interaction with salivary proteins. While these measurements demonstrate binding interactions and reveal differences in binding behavior among the saliva fractions, they do not provide information regarding the protein populations in saliva that are contributing to these interactions. SV-AUC was therefore employed to evaluate the changes in the sedimentation behavior of salivary protein populations as a result of quinine binding and thereby characterize the major sedimenting populations potentially involved in these interactions. 

### Characterization of Salivary Protein-quinine Interactions by SV-AUC

While SV-AUC is widely used to characterize macromolecular interactions, its application to human saliva research remains limited; partly owing to the compositional complexity and protein heterogeneity of the saliva matrix, and the predominant use of alternative techniques in similar studies to investigate salivary protein-polyphenol binding interactions in astringency perception (e.g., spectroscopy, chromatography, etc.) [55–57]. In the present study, SV-AUC was employed to characterize the major salivary protein populations involved in quinine interactions and to evaluate quinine induced changes in the sedimentation behavior of salivary proteins under native, physiologically relevant conditions and concentrations.

**Fig. 4:**
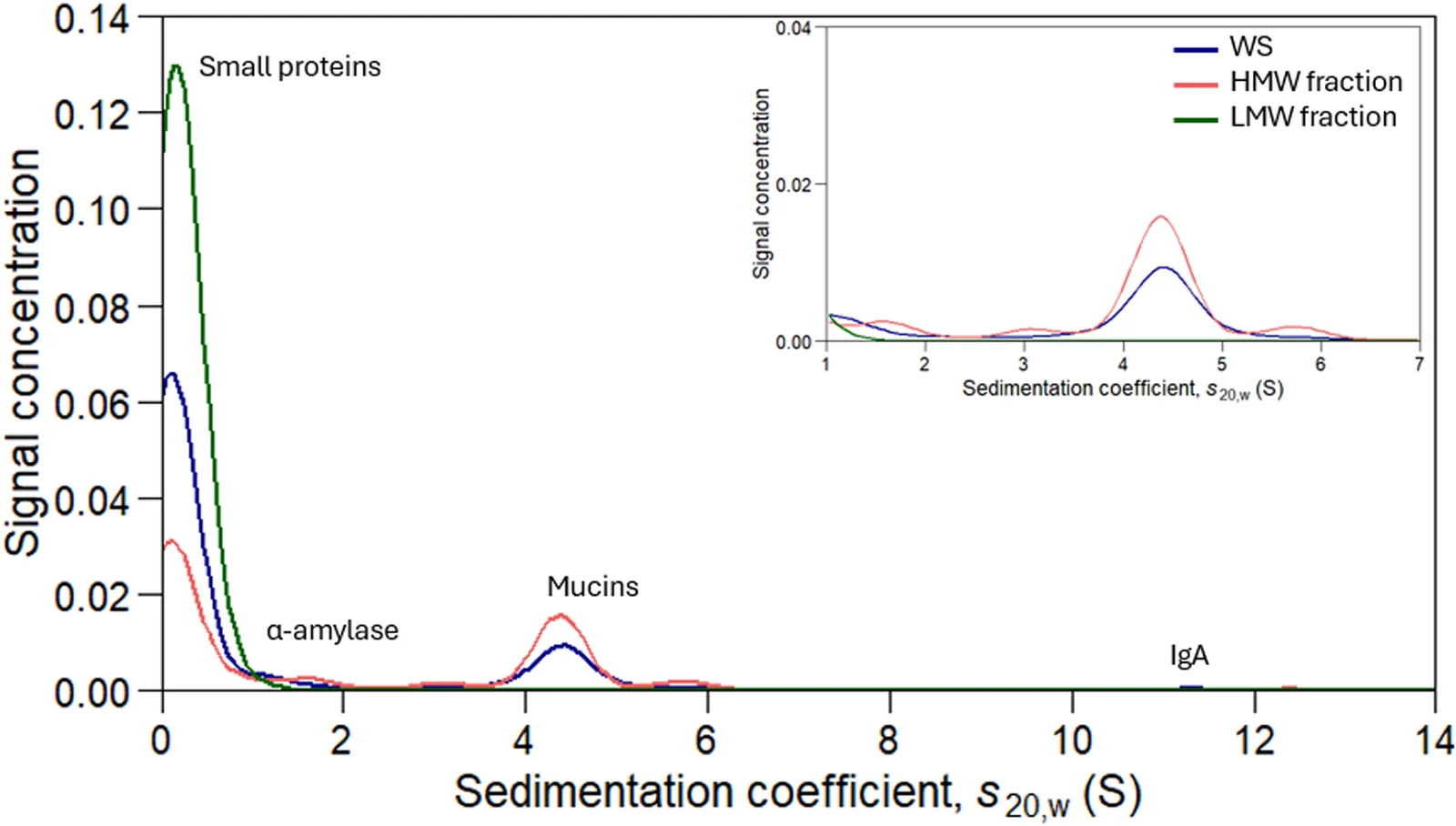
Sedimentation coefficient distributions for whole saliva (WS, dark blue), HMW fraction (red), and LMW fraction (green) containing 0.2 mg/mL protein. Distributions were obtained by fitting the Lamm equation solution in UltraScan III. The insert shows a magnified view of the sedimentation coefficient distributions from 1.0 to 7.0 S, with the Y-axis scaled to 0-0.04 for improved visualization of the peaks in this region. Peak assignments corresponding to major macromolecules were based on previously reported literature, while the peak at 0.09 S was tentatively assigned to small and low-molecular-weight proteins and peptides. All SV-AUC experiments were performed at 20 °C with a rotor speed of 60,000 rpm

Prior to examining salivary protein-quinine mixtures, the sedimentation coefficient distributions of WS, HMW fraction, and LMW fraction were characterized under the respective pH conditions (Fig. 4). In SV-AUC experiments, high rotor speeds such as 60,000 rpm used here are necessary for the detection of proteins and peptides below 10 kDa, as these small species sediment very slowly and produce diffusion boundaries at slower speeds, making them difficult to resolve [58, 59]. Quinine alone, analyzed under the corresponding solvent conditions, exhibited two sedimenting species centered at approximately 0.1 S and 0.18 S.

For WS, in the absence of quinine, the sedimentation coefficient distribution reveals two distinct peaks centered at approximately 0.09 S and 4.4 S. The peak centered at 0.09 S is tentatively attributed to small and low-molecular-weight proteins and peptides, while the peak at 4.4 S is consistent with the presence of higher molecular weight mucins. In addition, the declining shoulder of the 0.09 S peak exhibited a small peak centered at approximately 1.5 S, which was tentatively assigned to α-amylase. Lastly, a minor peak centered at 11.33 S was also observed and is likely attributable to immunoglobulin A (IgA). These assignments are broadly consistent with previously reported sedimentation coefficient (s_20, w_) values for salivary α-amylase, mucins, and IgA (1.5 S, 4 S, and 11 S, respectively) [60, 61]. Although these previous studies did not report a peak at low sedimentation coefficients, this difference, together with the minor variations in the remaining sedimentation coefficients, is likely attributable to differences in the saliva pools, sample collection and processing methods, experimental conditions, and data processing approaches. The assignment of the 0.09 S peak to small and low-molecular-weight proteins and peptides is supported by the work of Schuck et al. [62], who reported sedimentation coefficients below 0.6 for Peptide C and its fractions derived from human apolipoprotein C-II (molecular weights 2.0-3.5 kDa; 0.5 mg/mL), analyzed by SV-AUC at 40,000 rpm over 24 h. While these peptides are different from salivary proteins and peptides in terms of structure and composition, the reported sedimentation behavior supports that molecules with comparable molecular weight sediment at low S values, supporting the tentative assignment of the 0.09 S peak in the present study.

The HMW fraction displayed the same four peaks, with relatively greater signal concentrations associated with the larger macromolecular components, particularly mucins, compared to WS, as expected following enrichment of these proteins during fractionation. The mucin fraction of human saliva is comprised of 5 fractions: MUC1, MUC4, MUC5B, MUC7, and MUC19, with MUC5B and MUC7 being the predominant forms [63, 64]. The observed mucin peak in WS and HMW fraction is likely to represent a combination of these species. The peak at 0.09 S was also present in the HMW fraction, although with notably lower signal concentration than in WS, consistent with the removal of proteins and peptides smaller than 3 kDa during fractionation.

In contrast, the LMW fraction displayed a single predominant peak centered at 0.14 S and no other detectable peaks corresponding to α-amylase, mucins, or IgA, confirming the effective removal of these proteins by the 3 kDa MWCO filter. The slightly higher sedimentation coefficient of the peak corresponding to small proteins and peptides compared with the corresponding peak observed in WS and HMW fraction likely reflects differences in composition of the isolated protein population following fractionation.

**Fig. 5:**
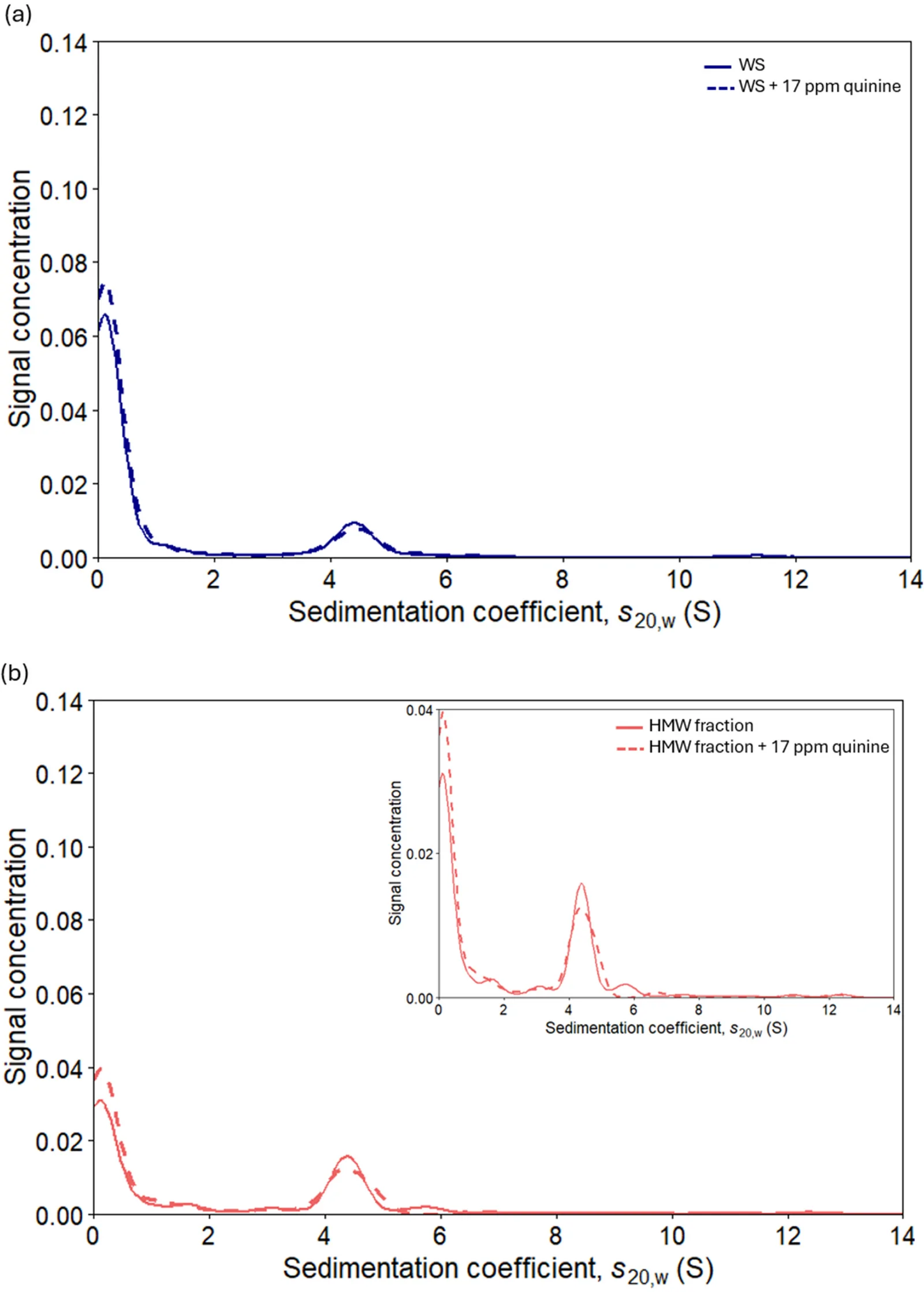

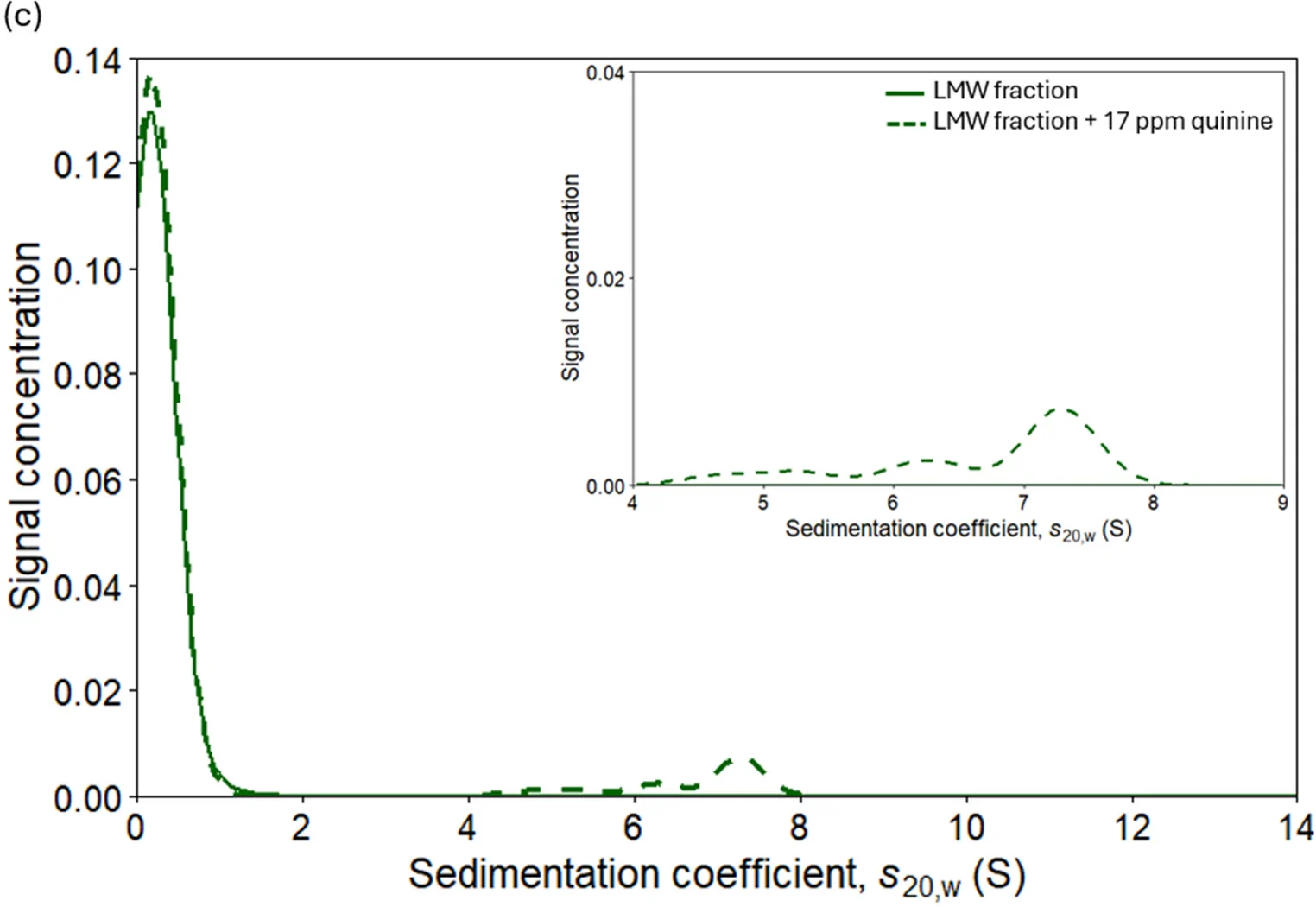
Sedimentation coefficient distributions of (**a**) whole saliva (WS), (**b**) HMW fraction, and (**c**) LMW fraction in the presence and absence of quinine. Solid lines represent respective salivary protein systems, and dashed lines represent the corresponding saliva-quinine system. The salivary protein concentration (0.2 mg/mL) was maintained constant in all samples, with pH values of 7.98, 8.33, and 8.03 for WS, HMW fraction, and LMW fraction, respectively. (**a**) Addition of quinine resulted in a reduction in the signal concentration of peaks corresponding to α-amylase, mucins, and IgA, consistent with the interaction of these salivary protein populations with quinine. (**b**) the HMW fraction exhibited similar reduction in major macromolecular peaks following quinine addition, accompanied with the appearance of new sedimentation species at higher sedimentation coefficient. (**c**) the LMW fraction exhibited the appearance of three new peaks at higher sedimentation coefficients following quinine addition, consistent with the formation of salivary protein/ peptide-quinine complexes. Inserts show a magnified view of (**b**) sedimentation coefficient distributions from 0 to 14.0 S with the Y-axis scaled to 0-0.04 for improved visualization of the peaks in the region and (**c**) the sedimentation coefficient distributions 4.0 to 9.0 S, with the Y-axis scaled to 0-0.04 for improved visualization of the peaks in this region. All SV-AUC experiments were performed at 20 °C with a rotor speed of 60,000 rpm

Figure 5a shows the changes in sedimentation coefficient distributions of WS following quinine addition. A subtle increase in signal concentration was observed for the peak centered at 0.09 S, accompanied by reductions in the signal concentrations of the peaks assigned to α-amylase, mucins, and IgA. The increase in signal concentration at this low sedimentation coefficient may partly reflect overlapping contributions from the small and low-molecular-weight proteins and peptides together with unbound quinine, which exhibited sedimenting species in similar low sedimentation coefficient region under the experimental conditions employed. In contrast, the reductions in the signal concentrations of the peaks corresponding to α-amylase, mucins, and IgA are consistent with the participation of these salivary proteins in binding interactions with quinine. Since both WS and WS + quinine systems contained the same total salivary protein concentration (0.2 mg/mL), the observed reductions in peak signal concentrations cannot be attributed to differences in salivary protein concentration between the samples. Similar observations were reported by Dinu et al. [61] for mixtures of whole human saliva (2.36 mg/mL) and epigallocatechin gallate (EGCG; 0.5 mg/mL), where a 47% reduction in the α-amylase peak area was attributed to the interaction between salivary α-amylase and EGCG.

Comparable changes were observed in the HMW fraction following quinine addition (Fig. 5b), with reductions in the signal concentration of peaks corresponding to α-amylase, mucins, and IgA, together with an increase in signal concentration at 0.09 S. In addition, the appearance of a new sedimenting species at a higher sedimentation coefficient (12.4 S) was observed, consistent with the formation of heavier protein-quinine complexes. These changes were more apparent than those observed in WS, likely reflecting the greater abundance of these proteins in the HMW fraction. Once again, the increase in signal at 0.09 S may reflect overlapping contributions from the smaller proteins together with unbound quinine, which sediments in a similar, low sedimentation coefficient region.

The strongest evidence of complex formation was observed in the LMW fraction (Fig. 5c). While this fraction exhibited a single peak centered at 0.14 S in the absence of quinine, in its presence there appeared three new sedimenting species centered at approximately 5.2 S, 6.23 S, and 7.3 S, together with an increase in signal concentration at 0.14 S that may reflect overlapping contributions from low-molecular-weight proteins and peptides and unbound quinine. The appearance of these higher sedimentation coefficient species following quinine addition provides evidence consistent with the formation of larger complexes between salivary proteins or peptides and quinine. The presence of multiple newly formed sedimenting species, rather than a single additional peak, further suggests the formation of complexes differing in size and/or stoichiometry, consistent with the heterogeneous composition of the LMW fraction and the varying capacities of individual proteins and peptides to interact with quinine. In particular, peptides below 3 kDa are generally more solvent-exposed than larger, folded proteins due to their limited tertiary structure, rendering a greater proportion of their amino acid side chains accessible for intermolecular interactions. This increased solvent accessibility may enhance their ability to bind quinine through hydrophobic, aromatic, and electrostatic interactions, thereby contributing to the diversity of complexes observed. The observed increase in sedimentation coefficient from approximately 0.14 S to a maximum of 7.3 S indicates a shift toward rapidly sedimenting species and is consistent with quinine-mediated formation of larger protein/peptide assemblies or complexes.

Collectively, the SV-AUC findings provide evidence consistent with the formation of protein-quinine complexes in all three salivary protein systems, with the LMW fraction exhibiting the most clearly resolvable evidence for formation of multiple higher-sedimenting complexes under the experimental conditions employed. These findings corroborate the fluorescence quenching results, which demonstrated concentration-dependent quenching of quinine fluorescence by salivary proteins and identified the LMW fraction as exhibiting greater quinine binding than the HMW fraction at comparable protein concentrations. To further characterize the thermodynamic properties underlying these interactions, ITC was subsequently employed.

### Interaction of Salivary Proteins with Quinine by ITC

ITC is a powerful biophysical technique that directly measures the heat energy absorbed or released during molecular binding events, thereby enabling thermodynamic characterization of protein-ligand interactions. It has previously been employed to study interactions between salivary proteins and polyphenols, predominantly in the context of astringency perception and using model protein systems. For example, Frazier et al. [65] studied the molecular interactions between hydrolysable tannins and the model globular protein bovine serum albumin; Poncet-Legrand et al. [66] employed poly(L-proline), a model proline-rich protein, to characterize its interactions with flavan-3-ols; Manjón et al. [67] used three high-molecular-weight salivary proteins – albumin, mucin, and α-amylase – to investigate interactions with oligomeric procyanidins; and Zhu et al. [68] used mucin-containing (0.2% w/v) artificial saliva to evaluate the thermodynamics of interactions with soya saponins, compounds responsible for astringency and bitterness of soybean products. Studies employing ITC with human saliva, or proteins derived directly from it, are considerably more limited; with a few examples being Ramos-Pineda et al. [69], who used pooled human saliva to study protein interactions with flavanol mixtures; and Soares et al. [70], who studied interaction of salivary proline-rich proteins with dietary tannins.

In the present study, ITC was used to characterize the thermodynamics of the interactions between salivary proteins and quinine. To the best of our knowledge, thermodynamic characterization of interactions between whole and fractionated human salivary proteins and a pure bitterant has not previously been reported.

**Fig. 6:**
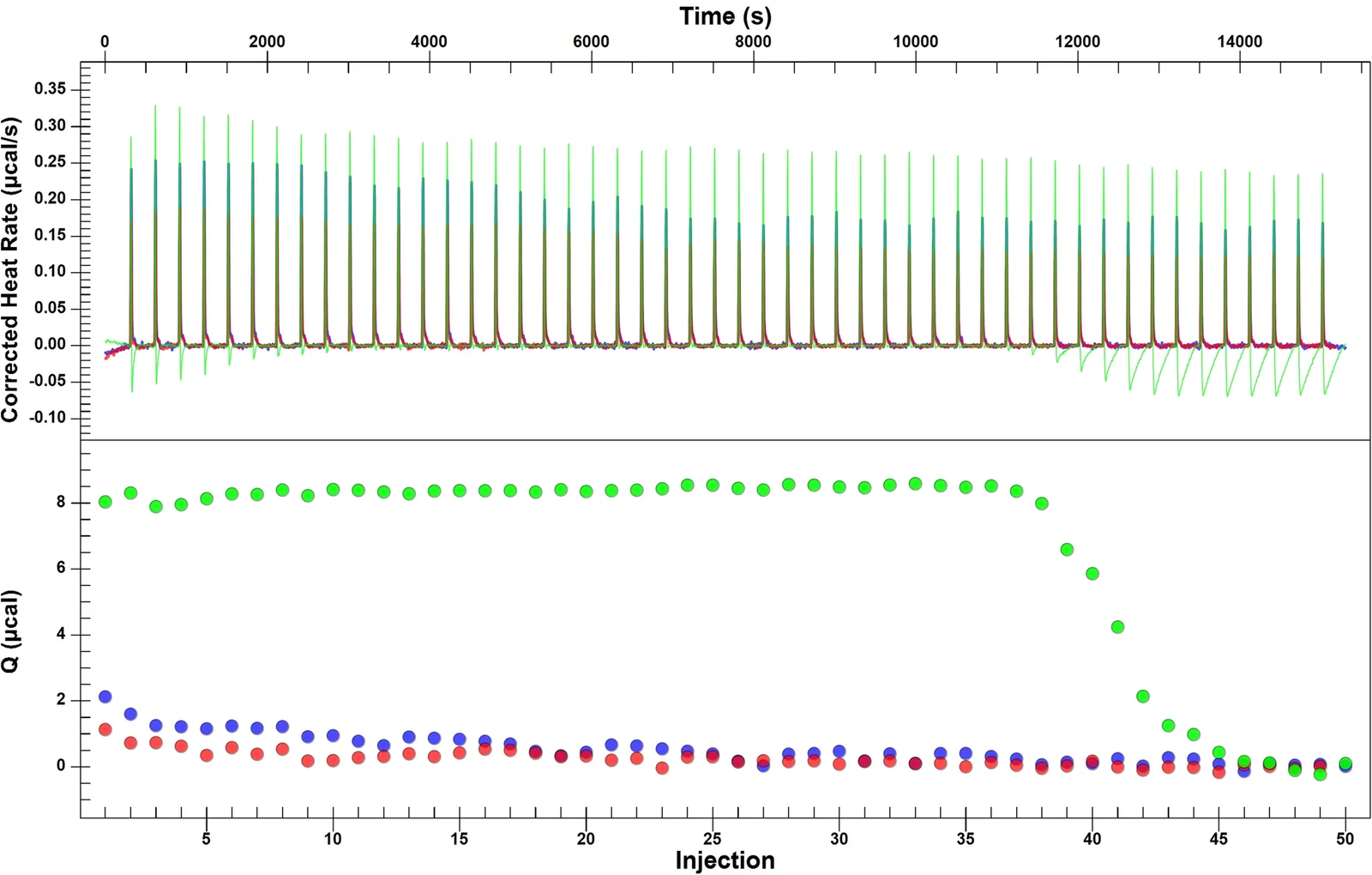
ITC profile of sequential injections of quinine (8 mM) titrated into whole saliva (blue), HMW fraction (red), and LMW fraction (green), all containing 0.2 mg/mL protein. Top panel represents dilution corrected heat rate (µcal/s) as a function of time. The lower panel shows integrated heat per injection Q (µcal) as a function of injection number

The ITC thermograms obtained upon titration of quinine into salivary protein solutions are shown in Fig. 6. Because the molecular weight, and consequently the molarity, of saliva cannot be determined with certainty due to its complexity and heterogeneous protein content, conventional ITC data analysis involving binding model fitting could not be performed to extract detailed thermodynamic fingerprints of these interactions, such as binding stoichiometry (n), association (K_a_) or dissociation (K_d_) constants, and entropy change (∆S) [71]. Nevertheless, an overlay of the thermograms (upper panel, Fig. 6) and the corresponding integrated heats for each successive injection (lower panel, Fig. 6) illustrate the relative binding trends between salivary proteins and quinine in the three systems. The resultant apparent binding enthalpies (∆H) of quinine to salivary proteins, corrected for the heat of dilution of quinine in water, are summarized in Table 2. The ITC thermograms reveal that interactions between salivary proteins and quinine produce positive apparent ∆H values across all three salivary protein systems, indicating a net absorption of heat during the reaction.

**Table 2:** Apparent binding enthalpies for the interaction of quinine with salivary proteins in different saliva systems, obtained from the integrated heats of successive injections using NanoAnalyze software for ITC. Values are reported as the mean ± 95% confidence intervals

Saliva sample	∆H (kcal/ mol of quinine)
Whole saliva (WS)	0.321 ± 0.548
HMW fraction	0.081 ± 0.158
LMW fraction	1.053 ± 0.009

It is instructive to consider multiple thermodynamic contributions that collectively constitute the observed heat signals, since the enthalpy measured by ITC is a global property of the system and thus reflects contribution from both the interacting species and the solvent [72]. In compositionally complex systems such as these, the measured enthalpic changes likely arise from a combination of: (1) heat changes associated with the formation of non-covalent interactions between salivary proteins and quinine, including hydrophobic, electrostatic, hydrogen bonding, and van der Waals interactions; (2) heat associated with self-aggregation among salivary proteins, quinine, or the protein-quinine complexes; (3) enthalpic contributions from quinine binding induced conformational changes in salivary proteins; and (4) heat arising from the disruption and reorganization of water molecules at the protein-quinine interface (desolvation) [69]. The observed positive enthalpy therefore reflects the net balance of favorable and unfavorable contributions from these individual processes [73].

Formation of non-covalent interactions, which is the primary driving force for protein-ligand binding, is generally enthalpically favorable and therefore exothermic in nature [74]. The positive ∆H values observed here suggest that these favorable contributions are outweighed by endothermic processes, most notably desolvation. Specifically, energy is required to disrupt the ordered water molecules surrounding both the salivary proteins and quinine as the molecules interact. Electrostatic interactions may occur between the protonated amine of quinine and acidic amino acid residues on the peptides. Hydrogen bonding involving the hydroxyl and methoxy groups of quinine may also contribute to the interaction. Given the hydrophobic nature of quinine, hydrophobic interactions with salivary proteins are also likely, with its quinoline aromatic ring system potentially participating in π–π interactions with aromatic amino acids such as phenylalanine, tyrosine, and tryptophan. A combination of these factors could promote reorganization of water molecules during binding, contributing to the observed endothermic enthalpy.

A similar observation was reported by Abraham et al. [75], who measured endothermic binding enthalpies of 6.3–6.5 kcal/mol upon isothermal titration of 10 mM zwitterionic and anionic phospholipid vesicles into 100 µM antimicrobial peptide GS14*D*k4 and attributed the positive enthalpy to desolvation effects that offset the exothermic heat associated with non-covalent bond formation. Jahmidi-Azizi et al. [76] characterized interactions between flavanols and salivary proline-rich proteins by titrating 10 mM catechin, epicatechin, or their mixture into pooled human salivary protein solutions (0.025 mM). Following fitting of a multiple independent sites binding model, the authors reported ∆H_1_ values of 7,300 ± 100 and 200 ± 60 cal/mol for catechin and epicatechin respectively, attributing this first entropy-driven binding component to hydrophobic interactions between the flavanols and proline-rich regions of the proteins. A second, enthalpy-driven binding component (∆H_2_ = -5,571 ± 8 and − 4,400 ± 80 for catechin and epicatechin respectively), was attributed to hydrogen bonding. Similarly, Zhu et al. [68] reported a weak endothermic interaction (∆H = 99.98 kJ/mol) upon titration of soyasaponin (~ 8.64 mM) into mucin solution (0.0017 mM), attributing the positive enthalpy to hydrophobic intermolecular interactions. The endothermic binding behavior observed in the present study is therefore consistent with previous reports of hydrophobically driven protein-ligand interactions in oral and flavor-related systems, however, other non-covalent interactions may also be contributing to the observed binding.

The apparent binding enthalpies differed notably across the three salivary protein systems (Table 2; Fig. 6). The LMW fraction exhibited the greatest apparent binding enthalpy (1.053 ± 0.009 kcal/ mol of quinine), with a narrow 95% confidence interval. In contrast, the apparent binding enthalpies for WS (0.321 ± 0.548 kcal/ mol of quinine) and the HMW fraction (0.081 ± 0.158 kcal/ mol of quinine) were smaller, with wider 95% confidence intervals that include zero. The relatively small and variable apparent enthalpic responses observed for WS and the HMW fraction may reflect the greater compositional complexity of these systems, including the presence of multiple protein constituents with potentially different interaction strengths and enthalpic contributions. These observations are broadly consistent with fluorescence spectroscopy and SV-AUC results (Figs. 3 and 5), which also indicated greater apparent interaction of the LMW fraction with quinine.

For additional context, the apparent binding enthalpy observed for NaCas under the same ITC conditions in a previous study from our laboratory [35] was 0.26 ± 0.02 kcal/ mol of quinine, determined by titrating 8 mM quinine into 0.8 mM NaCas. The apparent enthalpies observed for WS and the LMW fraction in the present study are numerically greater than those observed for NaCas. However, direct comparison of these values requires caution, as the experimental conditions differed in several aspects, including pH, protein concentration, expression of protein concentration (molar basis for NaCas versus mass-basis for saliva systems), and the overall composition of the protein systems. With these considerations, the greater apparent enthalpic response observed for the salivary systems, particularly the LMW fraction, is consistent with the greater quinine binding observed for salivary proteins relative to NaCas by fluorescence spectroscopy and provides an additional thermodynamic measure suggesting that, on a per-mass basis, salivary proteins may interact with quinine to a greater extent than the dietary protein sodium caseinate under the conditions studied.

Another important feature of the thermograms was the persistence of measurable endothermic heat throughout the titration, with the magnitude of heat per injection remaining virtually constant across most of the experiment. The LMW fraction was the only exception, exhibiting a progressive decline in the heat signal after approximately 38 injections, suggesting an approach toward saturation at higher quinine-to-protein ratios. In contrast, the heat signals observed for WS and HMW fractions remained relatively constant throughout the 50-injection titration, with no clear indication of saturation, consistent with weak, nonspecific binding. A similarly persistent heat signal throughout titration was reported by Abraham et al. [75], where the heat of consecutive injections remained uniform upon titration of antimicrobial peptide GS14Dk4 (100 µM) into phospholipid vesicles at concentrations of 10 and 33.4 mM.

In the present system, the persistence of measurable heat signal throughout the later stages of titration likely reflects both the compositional complexity of saliva and the heterogeneity of the proteins present. Different salivary proteins may possess binding sites with varying affinity for quinine, resulting in a broad distribution of binding strengths rather than a single, well-defined binding event. In addition, weaker interactions may require higher ligand concentrations before binding saturation can be approached [77].

Another possibility is that quinine binding induces conformational changes in salivary proteins, thereby exposing additional binding sites and sustaining the interaction beyond what would otherwise be expected. Barallat-Pérez et al. [78] reported that the addition of artificial saliva containing porcine gastric mucin (0.01% w/v) to food protein systems (whey, soy, or lupin proteins at 2% w/v) containing flavor compounds (C_6_ to C_10_ aldehydes and ketones at 5 mg/L) resulted in up to a 15-fold increase in protein-ketone binding and a 3-fold increase in protein-aldehyde binding compared with mucin-free protein-flavor systems. Binding was assessed from the headspace concentration of flavor compounds using headspace gas chromatography-mass spectrometry. The authors proposed that mucin-induced conformational changes in the protein exposed previously inaccessible hydrophobic binding pockets, thereby enhancing flavor binding, although the precise mechanism was not established. While the ligands and protein systems examined differ from those in the present study, this observation supports a broader notion that protein conformational changes upon ligand binding may also represent a potential factor responsible for sustaining or amplifying binding interactions.

The stronger interactions observed for the LMW fraction are also consistent with previous reports demonstrating the influence of protein composition on salivary protein-ligand interactions. Soares et al. [70] observed that the salivary P-B peptide (molecular weight ≈ 5.7 kDa) exhibited higher ITC-determined global binding constants toward several procyanidins (K_A_ = 1.08 × 10^11^ to 1.12 × 10^15^ M^− 2^) than the acidic proline-rich proteins (molecular weight ≈ 14.6 kDa; K_A_ = 5.4 × 10^7^ to 0.29 × 10^11^ M^− 2^). The authors attributed these differences to the amino acid composition of the P-B peptide, including its high proline content (~ 50% of the peptide sequence) and the abundance of hydrophobic amino acid residues, such as phenylalanine, leucine, and isoleucine, which were proposed to promote hydrophobic interactions and hydrogen bonding with procyanidins. Although the ligands examined differ structurally from quinine, these findings suggest that differences in salivary protein composition may contribute to the stronger interactions observed for the LMW fraction in the present study.

Collectively, and while acknowledging the differences in solution conditions, the ITC findings provide thermodynamic evidence consistent with the interactions observed by fluorescence spectroscopy and SV-AUC, with the LMW fraction showing the clearest overall evidence of interaction with quinine across the three techniques.

## Conclusion

This work investigated the interactions between the proteinaceous components of human saliva and the bitter compound quinine, using three complementary biophysical techniques: fluorescence spectroscopy, sedimentation velocity analytical ultracentrifugation, and isothermal titration calorimetry. Together, the findings provided converging evidence that salivary proteins interact with quinine, resulting in measurable enthalpic changes and the formation of protein-quinine complexes. The LMW fraction, enriched in proteins and peptides < 3 kDa, exhibited greater apparent quinine binding than the HMW fraction on a per mass of protein basis. This was evidenced by (i) a greater reduction in the concentration of unbound quinine determined by fluorescence spectroscopy, (ii) the appearance of new sedimentating species with higher sedimentation coefficients detected by SV-AUC, and (iii) a greater enthalpy of binding interaction measured by ITC. In contrast, the HMW fraction showed only a small reduction in unbound quinine concentration under the experimental conditions used for fluorescence spectroscopy, although the involvement of its constituent proteins in quinine binding was supported by reduced signal concentrations of mucins, α-amylase, and IgA in SV-AUC, together with the measurable heat of interaction quantified by ITC. These observations collectively highlight a potentially disproportionate contribution of the small and low-molecular-weight proteins and peptides to quinine binding in whole saliva and suggest that these constituents warrant further investigation.

Although the LMW fraction displayed greater apparent quinine binding than the HMW fraction on a protein mass basis, the specific constituent proteins and peptides responsible remain unidentified. Differences in protein composition, amino acid sequence, the number and accessibility of potential binding sites, and solvent conditions are among the factors that may have contributed to the observed differences in binding behavior.

In extant literature, salivary proteins have long been recognized for their interactions with dietary polyphenols and their contributions to astringency perception. In contrast, far less is known about their binding interactions with bitter compounds and the potential role such interactions may play in taste perception. Indirect evidence suggests a relationship between salivary protein composition and bitterness perception, with studies reporting a negative correlation between salivary protein concentration and bitterness perception in beer [79]; and a relatively higher abundance of proteolytically generated fragments of salivary amylase, immunoglobulins, and serum albumin in individuals hypersensitive to caffeine bitterness [12]. However, direct biophysical evidence supporting protein-bitterant interactions remains understudied, and the present work addresses this gap. To the best of our knowledge, this study represents the first direct biophysical assessment of interactions between whole and fractionated human salivary proteins and a pure bitterant, providing foundation for future investigations aimed at establishing the mechanistic links between salivary protein composition and bitterness perception.

The present study has some limitations that need to be considered when interpreting these findings. First, saliva collected from multiple healthy donors was pooled prior to analysis; while necessary to ensure adequate sample volumes for biophysical characterization, this step eliminates inter-individual variability in the salivary proteome. As a result, the extent to which biological differences in salivary protein concentration and composition, including differences in the abundance of LMW proteins and peptides, may influence quinine binding ability remains unknown, and warrants additional work in the future. Second, since the exact identity of the individual proteins and peptides within each fraction was not determined, protein concentrations were expressed on a mass basis rather than a molar basis. This precluded the determination of detailed binding parameters through conventional ITC model fitting and limited the thermodynamic characterization of the binding interactions to apparent binding enthalpy alone. Third, the fluorescence-based quantification of unbound quinine, while providing a practical estimate of apparent binding under the conditions studied, relies on calibration assumptions that may not fully account for the complexity of the salivary matrix, including contributions from dynamic or static quenching, differences in quinine protonation, and interactions with non-protein salivary constituents. Future work involving physical separation of protein-unbound quinine, along with the identification of specific salivary proteins involved in quinine binding through proteomics-based approaches will thus be important for determining the molecular factors underlying the observed differential engagement in quinine binding. Finally, the influence of these binding interactions on bitter taste perception and receptor activation was outside the scope of evaluation in present study, and the psychophysical implications of these findings remain to be established. Thus, the salivary protein-quinine complexation and associated enthalpic changes reported in the present work highlight the need for future studies aimed at determining the influence of salivary protein concentration and composition on individual differences in bitterness perception, whether such protein-bitterant interactions represent an important factor in bitter taste signaling, and the approaches that could be employed to modulate these interactions as a strategy to reduce perceived bitterness from food and pharmaceutical compounds.

## Data Availability

The data are available from the corresponding author on reasonable request.
